# Factors influencing the development of nursing professionalism: a descriptive qualitative study

**DOI:** 10.1186/s12912-024-01945-6

**Published:** 2024-04-26

**Authors:** Xingyue He, Ya Mao, Huili Cao, Linbo Li, Yanming Wu, Hui Yang

**Affiliations:** 1 School of Nursing, Shanxi Medical University, Taiyuan, 030001 China; 2 Department of Nursing, Linfen Hospital Affiliated to Shanxi Medical University (Linfen People’s Hospital), Linfen, 041000 China; 3 Department of Nursing, The First Hospital of Shanxi Medical University, Taiyuan, 030001 China

**Keywords:** Nurses, Nursing professionalism, Factors, Thematic analysis, Qualitative study

## Abstract

**Background:**

The shortage of nurses threatens the entire healthcare system, and nursing professionalism can improve nurse retention and enhance the quality of care. However, nursing professionalism is dynamic, and the factors influencing its development are not fully understood.

**Methods:**

A qualitative descriptive study was conducted. Using maximum variation and purposive sampling, 14 southern and northern China participants were recruited. Semi-structured interviews were conducted from May 2022 to August 2023 in face-to-face conversations in offices in the workplace or via voice calls. The interviews were transcribed verbatim and analyzed via thematic analysis.

**Results:**

Three main themes emerged: (1) nourishment factors: promoting early sprouting; (2) growth factors: the power of self-activation and overcoming challenges; and (3) rootedness factors: stability and upward momentum. Participants described the early acquisition of nursing professionalism as derived from personality traits, family upbringing, and school professional education, promoting the growth of nursing professionalism through self-activation and overcoming challenges, and maintaining the stable and upward development of nursing professionalism through an upward atmosphere and external motivation.

**Conclusion:**

We revealed the dynamic factors that influence the development of nursing professionalism, including “nourishment factors”, “growth factors”, and “rootedness factors”. Our findings provide a foundation for future development of nursing professionalism cultivation strategies. Nursing administrators can guide the development of nurses’ professionalism from many angles according to the stage they are in, and the development of professionalism deserves more attention. In the future, we can no longer consider the development of nursing professionalism solely as the responsibility of individual nurses; the power of families, organizations, and society is indispensable to jointly promoting nursing professionalism among nurses.

**Supplementary Information:**

The online version contains supplementary material available at 10.1186/s12912-024-01945-6.

## Introduction

The number of nurses leaving hospitals has been increasing, and the shortage of nurses is a significant problem faced globally [1, 2]. According to earlier studies, professionalism improves nurses’ clinical performance [3] and positively affects their adaptability (Park et al. 2021), reducing nurses’ burnout and turnover rates. Therefore, fostering professionalism in nurses and the factors that influence the development of professionalism are essential to producing effective nurses.

## Background

Nurses comprise the largest group of healthcare providers [4]. Nurses are a vital part of the healthcare system, with 27.9 million caregivers worldwide, according to the World Health Organisation’s Global Status of Nursing Report 2020 [5]. However, an unbalanced number of nurses and patients, high work pressure, and the fact that nurses face patients’ suffering, grief, and death each day have exacerbated burnout and led to the resignation of many nurses [6]. The COVID-19 outbreak has further exposed the shortage of nursing staff, especially in low- and middle-income countries where the scarcity of nurses remains acute. The lack of nurses not only has direct negative impacts on patients but also poses a threat to the entire healthcare system.

Nursing professionalism is closely associated with nurse retention and nursing practice [7–9]. Nursing professionalism is defined as providing individuals care based on the principles of professionalism, caring, and altruism [6]. As a belief in the profession, nursing professionalism is a systematic view of nursing that represents the practice standards and value orientation nurses utilize [10, 11]. According to previous research, nursing professionalism can enhance nurses’ clinical performance and positively impact their adaptability, reducing job burnout and turnover rates [6]. Furthermore, as nurses are the ones who provide “presence” care, cultivating nursing professionalism among nurses can promote interactions between nurses and patients, further improving the quality of nursing care and patient outcomes and injecting new vitality and hope into the entire healthcare system [12].

However, nursing professionalism is dynamic, and the cultural context also shapes nursing professionalism to some extent, leading to ambiguity in the factors influencing nursing professionalism. Initially perceived as mere “caregivers,” nurses have transformed into “professional practitioners,” emphasizing the nursing field’s seriousness and distinct professional characteristics [13, 14]. Nursing professionalism is also the foundation for developing the nursing profession [15]. Focusing on the factors influencing the development of nursing professionalism is one of the essential elements in providing an optimal environment for nurses’ professional growth and development in clinical practice [16]. Although some scales, such as the Hall Professionalism Inventory (HPI) [17], Miller’s Wheel of Professionalism in Nursing (BIPN) [18], Hwang’s Nurse Professional Values Scale (NPVS) [19], and Fantahun’s Nursing Professionalism Questionnaire [20] have been used to measure factors influencing the awareness, attitudes, and behaviors, they have their limitations. They struggle to encompass professionalism’s multidimensionality and complexity fully, overlook multilayered background factors, are constrained by standardization issues, may not account for individual differences, and often fail to capture dynamic changes over time [21–23].Compared with quantitative research methods, qualitative research can provide insights into the “unique phenomenology and context of the individual being tested,” which can help the researcher stay close to nurses’ professional lives during the research process and understand the personal, familial, and societal factors that influence nursing professionalism [24].Additionally, the understanding of nursing professionalism varies across different cultural and social contexts. In Western countries, research on nursing professionalism tends to incorporate professionalism across the entire nursing industry. In contrast, within China, research on professionalism tends to focus more on the individual level, with less attention to the perspectives of groups or the industry [25]. Therefore, through qualitative research, we can present nursing professionalism in a deeper, more affluent, and more transparent manner. Secondly, it is more authentic to understand the factors influencing nursing professionalism by directly obtaining relevant information from the perspective of nurses through dialogue with research participants as mutual subjects.

Given these considerations, we aim to answer the question of what factors influence the development of nursing professionalism. To provide more targeted strategies and recommendations for optimizing the nursing professional environment, enhancing nurses’ job satisfaction, improving t nursing quality, and contributing sustainably to patients’ and nurses’ health and well-being.

## Methods

### Aims

To explore the factors influencing the development of nursing professionalism. By incorporating nurses’ perspectives, we aim to improve our understanding of professionalism as individual, family, and socio-cultural influences. With this knowledge, we can inform strategies for developing nursing professionalism.

### Study design

A descriptive qualitative approach was adopted based on naturalistic inquiry [26, 27] and analyzed using the thematic analysis method described by Braun and Clarke [28]. Semi-structured interviews were conducted between May 2022 and August 2023 with nurses in southern and northern China hospitals. Furthermore, the research findings were reported in accordance with the consolidated criteria for reporting qualitative research (COREQ) (Supplementary Material S1) [29].

### Participants and settings

We chose hospital nurses as study participants based on considerations of their nursing experience. Firstly, the Chinese government has implemented a policy of accountable holistic care, whereby registered nurses take on the entire cycle of a patient’s physical, mental, and spiritual care [30]. Secondly, new nurses must undergo two weeks to one month of basic training and a 12–24 month specialty rotation (for most new nurses who graduated before 2016, their training was completed by their departments). During this time, they are under the supervision of a superior nurse for holistic and responsible care [31].

We used maximum variance purposive sampling to recruit a heterogeneous sample of information-rich key participants [32]. Participant selection considered variations in role classification, years of experience, and educational levels of Chinese nurses [33]. The purposive variation allowed the discovery of Chinese nurses’ unique perceptions of nursing professionalism. Inclusion criteria: (1) registered nurses (providing direct services to patients within the unit), nurse managers (directly supervising and guiding the clinical work of registered nurses), nursing department managers (managing nurse managers throughout the hospital), with at least one year of nursing experience; (2) voluntary participation. Exclusion criteria: (1) nurses not working during the hospital’s study period (holidays, maternity leave, or sick leave); (2) refresher nurses.

### Data collection

The same researcher conducted each interview to ensure consistency. Before the interviews, the interviewers systematically conducted in-depth theoretical research on relevant studies. The interviewer received guidance from professors with rich experience in qualitative research and undertook practice interviews to improve her interviewing skills. Interviewers encouraged interviewees to talk freely about their perceptions and used an interview guide (Supplementary Material S2), which was based on the findings of previous research on the conceptual analysis of nursing professionalism [6]. The questions were open-ended and general; ample space was left between questions to respond to interviewees’ comments. Semi-structured interviews began with a brief introduction to the topic (e.g., definition and explanation of nursing professionalism). Although the interviewer had an agenda for discussion, this format allowed the interviewee to deviate from this agenda and direct follow-up questions [34].

All interviews were conducted from May 2022 to August 2023 in face-to-face conversations in offices in the workplace or via voice calls and lasted between 35 and 94 min. Participants were asked to complete the main demographic questionnaire at the end of the interviews. The researcher recorded participants’ expressions, body language, and pauses during the interviews. Memos written by the researcher during the study were also used as analytical material.

### Data analysis

For rigorous qualitative sampling and data saturation, Braun et al. [35] propose that qualitative researchers require a sample appropriate to the research questions and the theoretical aims of the study and that can provide an adequate amount of data to answer the question and analyze the issue entirely. We reached thematic saturation after 14 interviews when no new codes or themes emerged.

A thematic analysis approach was used, following the phases described by Braun and Clarke [28]. The analysis comprised six stages: (1) immersing in the data; (2) creating initial codes; (3) identifying themes; (4) reviewing; (5) defining and labeling these themes; and (6) finally, composing the analysis report. Two researchers transcribed and analyzed the textual data. In the first stage, the researchers carefully read the interview transcripts to familiarize themselves with the depth and breadth of the content. In the second stage, preliminary codes were generated based on the research questions, initial interpretation of the data, and discussion of initial emerging patterns. At this stage, ensuring that all actual data extracts were coded and organized within each code was necessary. In addition, the following principles were used as guidelines: (1) code for as many potential themes/patterns as possible; (2) code extracts of data inclusively, i.e., preserve small sections of the surrounding data when relevant; and (3) code individual extracts of data for as many different “themes” as appropriate [36]. In the third phase, the two authors analyzed the initial codes, sorted them into potential themes, and debated their meanings and emerging patterns to reach a consensus. This phase, which refocused the analysis on the broader level of themes rather than that of codes, involved sorting the different codes into potential themes. In the fourth stage, reviewing themes ensures that the data supports the themes and allows an iterative process between different levels of abstraction without losing grounding in the raw data. Finally, defining the “essence” of each theme during the development of the main themes by identifying the “story” as consistent with the data and the research question while ensuring that the themes did not overlap but still fit together in the overall “story” of the data. It told the “story” by writing analytical narratives with illustrative quotes.

### Rigour

This study achieved credibility by selecting a heterogeneous sample, performing member checks, and taking field notes [37]. This study ensured dependability by verifying the findings with the researchers and participants, appropriately numbering the direct quotations (e.g., DN1), and comparing the results with the previous literature. This study established confirmability via audit trails [38] and the comprehensive reporting of all research processes. This study ensured transferability by describing the data collection process and seeking a heterogeneous sample.

## Findings

**Fig. 1:**
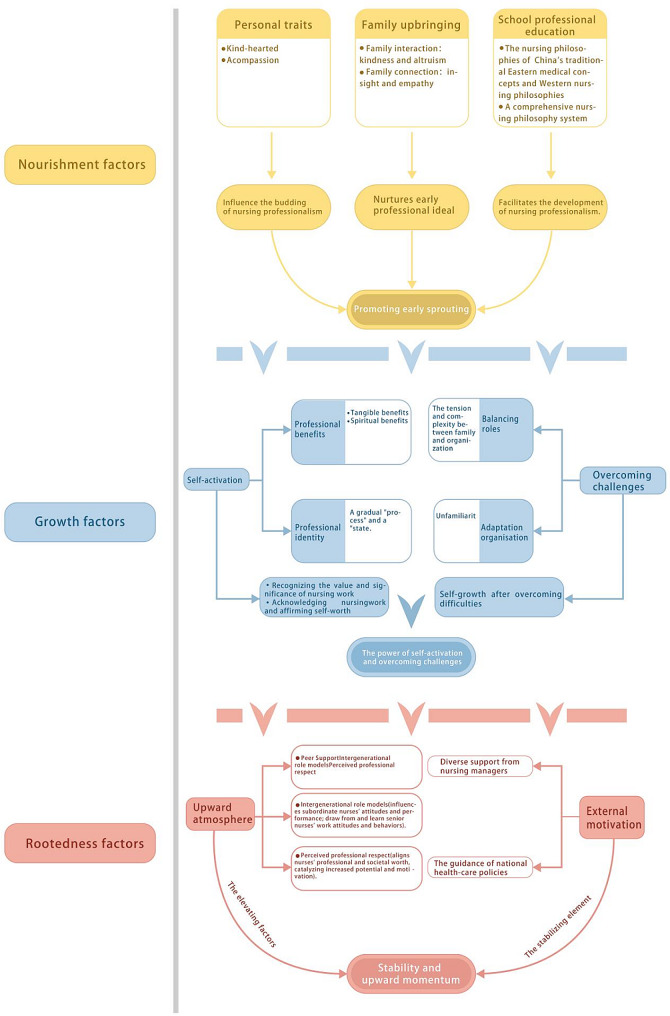
Factors influencing the development of nursing professionalism

Fourteen participants were interviewed (the demographic information is presented in Table 1). The thematic analysis identifies three major themes (Fig. 1). These interconnected topics illuminate the growth process and factors influencing nursing professionalism. The first theme, “nourishment factors: promoting early sprouting,” includes personal traits, family upbringing, and professional education at school and emphasizes early factors influencing nursing professionalism. The second theme, “growth factors: the power of self-activation and overcoming challenges,” included self-activation and overcoming difficulties, focusing on the dual attributes of the growth process of nursing professionalism. The final theme, “rootedness factors: stability and upward momentum,” includes an upward atmosphere and external motivation and explores the factors that maintain the stability and sustainability of nursing professionalism.

**Table 1:** Characteristics of participants interviewed

Participants	Number	Gender	Age	Education	Average interview duration (Minutes/times)	Length of Service	Geographic location
Nursing department managers	ND1	Female	63	Master	60/1	44 years	Northern China
	ND2	Female	32	Master	94/1	10 years	Northern China
	ND3	Female	60	Master	46/1	40 years	Northern China
Nurse managers	NM1	Female	40	Doctor	35/1	18 years	Northern China
	NM2	Female	42	Master	62/1	20 years	Northern China
	NM3	Female	34	Bachelor	72/1	12 years	Northern China
Registered nurses	N1	Female	37	Master	60/1	15 years	Northern China
	N2	Female	36	*Vocational Education	67/1	18 years	Northern China
	N3	Female	28	Master	90/1	1 year	Northern China
	N4	Female	29	Doctor	82/1	1 year	Southtern China
	N5	Female	28	Master	38/1	3 year	Northern China
	N6	Female	32	Master	50/1	7 year	Northern China
	N7	Female	64	*Vocational Education	88/1	45 years	Northern China
	N8	Female	34	Bachelor	81/1	12 years	Southtern China

*Vocational Education: the initial level of nursing education in China

### Nourishment factors: promoting early sprouting

#### Personal traits

Personal traits are called “nature” [23]. There exists a close connection between personal traits and professional behavior. When nurses confront patients’ physiological and emotional needs, innate qualities like kindness and compassion predispose them to be more sensitive to patients’ suffering and needs. Nursing professionalism transcends mere task fulfillment; this inner emotional drive compels nurses to fulfill their duties and engage in nursing work out of a genuine desire and sense of responsibility, practicing the nursing mission nobly. Thus, whether individual traits align with the nursing mission profoundly influences the nursing professionalism of nurses in their work.

*“At 32, I became a head nurse, full of vitality and boundless enthusiasm, particularly compassion. I have no idea where this compassion comes from.” (ND1)*.

#### Family upbringing

Education begins in the family, and it is through family education that nurses develop an early sense of professionalism. China has a “family culture” that defines the responsibility of family education. The study participants recalled that in childhood, the “living” nature of family education shaped early professionalism, in which the concepts of “kindness” and “altruism” were acquired through interactions with family members.

*“My mother was an early childhood educator, and when she told me fairy tales, it was to promote kindness. Loving others and being selfless, you can’t be a bad person. That’s what altruistic education is about.” (ND1)*.

The impact of family education on the acquisition of nursing professionalism extends into adulthood. In Chinese Confucianism and collectivism, family members usually have close emotional ties, and this “strong bond” family structure promotes nurses’ understanding and care for others and their ability to be wiser and more caring in the nursing profession. This strong bonding plays a catalytic role in the emergence of nursing professionalism.

*“Some nurses are very adept at expressing care, perhaps because grandparents and parents live together. Since childhood, parents have taught them how to express care.” (N4)*.

#### School professional education

Nursing professionalism is further acquired through professional education in schools. Nursing professional education emphasizes respect and care for patients, adherence to social responsibility, and the integration of traditional Chinese oriental medical thought and Western nursing concepts, internalized into behaviors to form the concept of professional nursing spirit. Participants indicated that the virtues of dedication, responsibility, respect, and caring that permeate school professional education are incorporated by nurses into nursing practice.

*“The best nursing comes from the heart. When I was administering injections, I thought about how to alleviate the patient’s pain. Later, I learned that if I entered the needle quickly, it would be less painful, so I often practiced in the operating room.” (N8)*.

Other participants also shared that they felt positively guided by professional education at school, constructing a comprehensive nursing philosophy system within the educational context. They realized that nursing is a multidisciplinary field encompassing human care, social responsibility, and ethical values.

*“University was my most unforgettable learning experience. I studied 36 courses here, including nursing aesthetics, literature, sociology, ethics, education, etc. I realized that the nursing work we engage in has such rich depth! has become an invaluable treasure in my nursing career.” (ND1)*.

### Growth factors: the power of self-activation and overcoming challenges

#### Self-activation

##### Professional benefits

Professional benefit perception refers to the advantages nurses perceive while engaging in nursing work, acknowledging that their involvement in nursing promotes their holistic personal growth [39]. Consistent with traditional perspectives, this study finds that nurses generate a sense of professional benefit through both “tangible benefits” and “spiritual benefits,” recognizing the value and significance of nursing work, thereby furthering the development of nursing professionalism.

The dynamic updating achieves “tangible benefits.” Nurses require outstanding professional competence and ongoing continuing education. Participants mentioned that nurses utilize their professional knowledge and clinical experience to save patients’ lives, and exceptional professional competence can rekindle their enthusiasm for work. Continuous and dynamic continuing education, supplementing the latest technology and knowledge in the nursing field, can generate positive professional emotions.

*“There’s only one doctor on duty at night, and nurses are the first responders when we encounter emergencies. Even before the doctor arrives in the ward, I must act quickly and urgently. Every time I bring a patient back from the brink of death, I feel excited throughout the night.” (N6)*.

“Experience is und*oubtedly important. I’ve been working for over a decade, and I undergo training every year. No one likes stagnation; we can forge ahead only by continually moving forward.” (NM3)*.

Self-worth realization through “spiritual benefits.” Experiencing a sense of value in nursing practice provides nurses with positive reinforcement, enhancing nursing professionalism behavior. Moreover, as healthcare practitioners, the ability of relatives and family members to benefit from it distinguishes Chinese nurses’ unique approach to self-worth realization from nurses in other countries. This unexpected feedback, whether in material or spiritual forms, enables nurses to fulfill their sense of worth.

*“Sometimes, friends and relatives ask me about hospitalization-related questions, and I am more than willing to help them.” (N2)*.

*“ I changed my mother’s gastric tube without any complications.” (NM3)*.

##### Professional identity

Nursing professional identity refers to nurses acknowledging their work and affirming their self-worth [40]. This study defines professional identity as a gradual “process” and a “state.”

One participant mentioned that professional identity is a psychological “process” that nurses develop and confirm their professional roles through their personal experiences. It is closely related to the individual experiences of nurses. Nurses’ gradual recognition of their work prompts them to progress and develop a positive work attitude and professionalism.

*“Gradually, I discovered that being a nurse makes me realize my significance, which keeps me moving forward, time and time again.” (ND2)*.

Simultaneously, as a “state,” professional identity represents the degree to which nurses identify with the nursing profession. This “state” of professional identity reflects nursing professionalism’s long-term accumulation and formation. It indicates nurses’ long-standing dedication and emotional involvement in nursing, leading to higher professional competence and a sense of responsibility in their work.

*“It’s not just a job to make a living; it’s about wholeheartedly identifying with this profession, unleashing one’s potential, which results in better professional conduct.”(N5)*.

#### Overcoming challenges

##### Balancing roles

Balancing roles refers to the equilibrium individuals establish between their roles in the nursing profession, family, and organization. Nursing professional roles are inherently multifaceted, and when faced with multiple responsibilities, such as family demands and organizational tasks, nurses must balance these roles. The tension and complexity between personal and organizational roles can potentially inhibit their emotions and professional motivation. However, in China, families are tightly knit, and strong family support can help reconcile this tension.

*“To be a good nursing department manager, you need strong family support. The commitment to one’s career and the dedication to family don’t always align. For instance, my job keeps me busy regarding family matters, and I have limited time to care for my children. My parents-in-law take care of them more. I do rounds every Sunday, and the phone never stops ringing, even on my days off. There’s no way around it; this is the role I’ve taken on. Family support allows me to work with peace of mind.” (ND3)*.

##### Adaptation organization

Nurses also face challenges in adapting to organizational systems. These adaptability challenges include rapidly learning new technologies, processes, and the culture of practice in different departments. This “unfamiliarity” impedes the manifestation of nursing professionalism. Participants indicated that the inability to adapt to clinical work quickly affects new graduate nurses’ transition into practice. Initially, there is a “honeymoon period” when becoming a registered nurse, but as actual capabilities do not align with expected performance, the excitement gradually wanes.

*“I didn’t know the routine procedures in ophthalmology, I couldn’t measure eye pressure, and I didn’t know how to perform eye injections. I was terrified, which brought various challenges when I started working.” (N4)*.

Furthermore, nurses must adapt to the practice culture of “this is how things are done” and “it’s always been done this way” in their workplace. Due to the promotion and title system requirements in Chinese hospitals, nurses with several years of experience often need to rotate through departments such as Intensive Care Unit and emergency for a period. The differences in operations and management between different departments also frustrate these nurses during rotations. However, a certain social prestige is attached, making it challenging for the nurses from the original department to provide direct guidance to the rotating nurses, leading to isolation for the latter in new departments.

*“A blank slate regarding the department’s hierarchy, administrative procedures, and so on.” (N3)*.

*“Although there’s a set of procedures, mostly similar, it’s the slight differences that always set me apart.” (N6)*.

### Rootedness factors: stability and upward momentum

#### Upward atmosphere

##### Peer support

Peer support has a positive impact on nursing professionalism. Peers are individuals of the same age group who have formed a connection due to shared experiences in similar socio-cultural environments, with emotional support, mutual assistance, and understanding constituting the core elements of peer support [41]. Firstly, nursing work often involves highly stressful situations, including heavy workloads, complex patient conditions, and urgent medical cases. Peer support provides emotional support, allowing nurses to find comfort and encouragement when facing stress and difficulties. Secondly, peer support cultivates a positive work atmosphere and team spirit. In a mutually supportive, trusting, and cooperative team, nurses are more likely to experience a sense of accomplishment in their work. They feel they are not isolated but part of a united and collaborative whole. Furthermore, peer support also promotes professional development and knowledge exchange among nurses. In an open and supportive team environment, nurses are more willing to share their experiences and knowledge, learn from each other, and grow.

*“The spirit influences the spirit, especially those of my age group who have left a deep impression on me with their admirable qualities in their work. It makes me reflect on my shortcomings in my work and constantly strive to improve and adjust myself.” (N2)*.

##### Intergenerational role models

Inter-generational refers to the relationships between generations [42]. In nursing practice, inter-generational relationships exist, such as those among nurses of different ages and levels of experience. This study’s inter-generational role models include managerial role models and senior nurses.

Participants believe that managers’ professionalism influences subordinate nurses’ attitudes and performance. The professionalism of managers not only plays a guiding and leadership role in daily work but, more importantly, sets an example, inspiring and encouraging subordinate nurses who are willing to follow and inherit professionalism.

*“The department’s leadership has a significant impact on professionalism. When managers have a strong sense of professionalism, the nurses they oversee follow suit. Because leadership represents the management level and higher things, it’s difficult for things at the bottom to go well if it’s not well-controlled at the top.” (N8)*.

On the other hand, senior nurses, as role models within the nursing generation, also significantly impact the upward development of professionalism. Senior nurses’ rich experience and professional competence guide new nurses to maintain a rigorous attitude at work. New nurses often draw from and learn senior nurses’ work attitudes and behaviors, catalyzing the elevation of nursing professionalism.

*“Senior nurses have a role model effect because new nurses learn from the older ones. If senior nurses work rigorously and new nurses make mistakes or lack a sense of dedication, they will immediately point it out. Over time, you also become more rigorous.” (NM3)*.

##### Perceived professional respect

Societal respect for nursing work creates an atmosphere of care and emphasis on nursing. Nurses within this atmosphere become aware of the importance of nursing work and the profound significance of patient care. They are inclined to exhibit positive nursing professionalism behaviors to meet the expectations of society and the general public.

*“The nursing industry has experienced the COVID-19 pandemic, and during the anti-epidemic efforts, nurses were at the forefront, risking their lives to care for patients, receiving acclaim from patients, doctors, and the public.” (N2)*.

Professional respect is the manifestation of nurses’ self-acknowledgment of nursing values. It is more than an external acknowledgment; it is an internal affirmation. This mutual respect aligns nurses’ professional and societal worth, catalyzing increased potential and motivation.

#### External motivation

The stability of nursing professionalism relies on external resources, including the diverse support from nursing managers and the guidance of national healthcare policies. Nursing managers are the frontline leaders who interact with nurses, and their support serves as a management tool and a direct means to sustain nursing professionalism. This multifaceted support encompasses economic incentives such as compensation and reward mechanisms. It extends to non-material motivations such as career advancement opportunities, adequate staffing, modern equipment provision, and fair and equitable treatment form crucial aspects of managerial support. Providing nurses with stable external support creates a space to focus on their professional mission and responsibilities, thus maintaining the stability of nursing professionalism.

*“Economic foundation determines the superstructure(spiritual world)).” (NM1)*.

Furthermore, the guidance of national healthcare policies serves as a beacon for the development of the nursing profession. At the national level, healthcare policies can regulate the organization and operation of healthcare systems and services, providing nurses with a more stable and favorable working environment. This environment allows nurses to fulfill their professional roles better and maximize their value. The environmental changes brought about by policy guidance offer nurses more favorable professional conditions, effectively promoting the upward development of nursing professionalism.

*“Government documents summarize the needs of our society, and nursing will continue to improve in the direction of policy guidance.” (NM3)*.

## Discussions

This study provides insights for understanding the factors that influence the development of nursing professionalism. We emphasize the themes of early nourishment factors that promote the emergence of nursing professionalism, growth factors associated with self-activation and overcoming challenges, and rootedness factors that stabilize upward, which reveal the dynamic factors that influence the development of nursing professionalism.

We added the early influence of personality traits, family upbringing, and school professional education in the development of nursing professionalism, which is similar to the pathway through which nurses’ foundational values are acquired [43, 44]. Building on previous research, we highlight the sequential order of socialization in family education and school professional education, with individual socialization within the family achieving individual socialization before school professional education, emphasizing the importance of intergenerational family transmission on the development of nursing professionalism [45]. Education commences within the family, a social organization with an educational function. China values its “family culture” and emphasizes defining parental responsibilities for family education based on blood relations. It is a common folk law in China that parents are regarded as the first teachers. In addition, Chinese society promotes Confucianism, which emphasizes instilling the concept of “self-improvement” through “educational living” [46], as mentioned in our study, the interpersonal interactions such as “altruism” and “caring” arising from family interactions can help nurses establish a deeper emotional connection with their patients. Therefore, future consideration could be given to incorporating programs that foster culture and emotions into professional education. Similar studies are necessary in East Asian countries and other countries with similar cultures to broaden the results of factors influencing nursing professionalism.

The growth of nursing professionalism requires real work scenarios. Our results present the dual factors of nursing professionalism upon entering the workplace. Regarding self-activation factors, we delve into the significance of “professional identity” and, for the first time from the perspective of Chinese collectivism, explain the unique influence of “professional benefits” on nursing professionalism. Our study aligns with previous research, viewing professional identity as an ongoing “process” [47]. By developing a professional identity, nurses can exhibit “stateful” self-satisfaction and self-motivation, contributing to their job satisfaction and professionalism [48]. The “professional benefits” involve integrating rational and emotional aspects. The “tangible benefits” of professionalism and technical competence at work lead to positive experiences and emotions among nurses. Nurses voluntarily invest more passion and energy in their work [49]. In addition, what sets our results apart is how Chinese nurses obtain ‘spiritual benefits,’ which come from the convenience of medical access that their relatives enjoy due to their work. Some studies have shown that “spiritual benefits” are more apparent among nurses aged 40 and above and those with higher professional titles [50]. The accumulation of clinical experience and the harmonious interpersonal relationships achieved through medical collaboration can help family members access reliable medical resources, leading to greater professional gain. This phenomenon is closely related to the collective consciousness of Chinese nurses, revealing that people are not always “self-interested and rational”; their behavior is influenced by more complex factors such as intuition, emotions, and attitudes [49].

In terms of the challenges faced, on the one hand, we emphasized the supportive role of intergenerational relationships in nurses’ work-family conflicts. Previous studies have shown that Chinese nurses perceive nursing work as a means to fulfill family responsibilities rather than the ultimate goal, reflecting a prioritization of family over work [51]. Consequently, nurses are more likely to resign during work-family conflicts, reallocating their resources from work to family [52]. Compared with previous studies, we found that China is a highly connected society, and multi-generational households are relatively common [53]. Hence, the importance of maintaining good intergenerational relationships cannot be ignored in Chinese society and culture, substantially impacting nursing professionalism. On the other hand, we reveal the underlying reasons for the restricted development of nursing professionalism among nurses during the transition period. Newly graduated nurses face negative experiences such as incompetence, lack of preparation, exhaustion, and disappointment in their work, hindering the development of nursing professionalism, which is especially evident in departments such as obstetrics and gynecology, ophthalmology, and emergency, where teaching hours for these specialties fall significantly below those for general internal medicine and surgical nursing [54]. The educational experiences of nurses are insufficient to meet clinical demands [55]. Moreover, this is compounded by differences in the structure and content of the 12–24 month “standardized training” for new nurses that has already begun in most cities in China, further exacerbating the experience of separation of new nurses from their organizations [56]. The development of rotational nurses is often neglected, and transfer systems are a mere formality [57]. Therefore, developing nursing adaptability and creating a supportive work environment should be incorporated into the content and structure of different organizational transition programs to make a positive work environment and promote nurses’ engagement, enhancing nursing professionalism.

It is worth noting that the rootedness factor involves individual, organizational, and societal dimensions. At the personal level, peer support and intergenerational role models integrate the demonstration of actual “peers” and “role models” with nurses’ self-awareness and agency to achieve upward mobility in nursing professionalism [58]. However, while peer support offers emotional and social cognitive consistency based on age, background, and learning experiences, it may lack experiential depth [59]. In contrast, intergenerational role models involving a “superior-subordinate” relationship can initially lead to “nurturing” relationships, potentially leading to lateral violence and bullying [60]. At the organizational level, our findings highlight that professional respect in the workplace is more relevant to nurses’ professionalism than social appraisal. Professional respect is the nurses’ perception of their subjective social status within the profession and an analysis of the social value associated with the nursing profession [61]. However, nurses are not always respected, especially as insults and disregard from patients, superiors, or physicians can lead to negative emotions, professional burnout, and a desire to quit [62–64]. Regarding the societal dimension, providing external motivation tailored to nurses’ specific backgrounds and needs is beneficial for the stable development of nursing professionalism. Financial incentives are often considered a common strategy to improve nurses’ motivation and retention in motivation management [65]. However, the effectiveness of incentives is, more importantly, dependent on the response of nurses after implementation, and it is crucial to understand the needs and preferences of nurses in terms of incentives as well as the level of nurses’ participation in policy development, in addition to material rewards [66, 67].Therefore, maintaining the stability of nursing professionalism is therefore complex, and nursing managers should consider ways to deepen peer support and reduce workplace bullying through “intergenerational parenting”, and should develop policies that support nurses, have zero-tolerance for disruptive behaviours, uphold the professional dignity of nurses, and ensure that their voices are heard and valued, which contributes to a more positive, fulfilling, and motivating nursing work environment.

## Limitations

Given the persistently low number of men in nursing, all participants recruited for our study were female. However, considering the relatively narrow focus of the research, The purposive variation, and the richness of the generated data, the sample size was deemed sufficient to achieve our objectives. In addition, although the study results reveal dynamic influences on the development of nursing professionalism, they do not differentiate between nurses at different career stages, such as novice and expert nurses. We consider these factors as “common characteristics” for them, intertwined with each other, which can be further clarified in future research.

## Conclusions

This study is an important addition to previous research in that we reveal the dynamics of factors that influence the development of nursing professionalism, including the “nourishment factor,” “growth factor,” and “rootedness factor.” Our findings provide contextual factors that can be changed during the development of nursing professionalism and lay the foundation for future strategies to foster nursing professionalism.

### Relevance to clinical practice

The findings of this study have important implications for exploring the development of nursing professionalism. Nursing managers can support nurses’ professionalism from various perspectives, depending on the stage of the nurse’s life, such as valuing nurses’ family relationships, focusing on nurses in transition, listening to nurses’ voices, and creating a “magnetic nursing” work environment. These measures will not only positively impact the careers of individual nurses but will also help improve the standard and quality of health care in general. In the future, we should no longer view the development of nursing professionalism as solely the responsibility of individual nurses; the influence of family, organizations, and society is indispensable in collectively promoting the development of nurses’ nursing professionalism.

### Electronic supplementary material

Below is the link to the electronic supplementary material.

Supplementary Material 1

## Data Availability

Data used to support the findings of this study are available from the corresponding author upon request.

## Abbreviations

HPI: The Hall Professionalism Inventory
BIPN: Miller’s Wheel of Professionalism in Nursing
NPV: SHwang’s Nurse Professional Values Scale
COREQ: The consolidated criteria for reporting qualitative research
