# Supplementary material for: Factors influencing the development of nursing professionalism: a descriptive qualitative study
Source: BMC Nurs. 2024 Apr 26;23:283. doi: 10.1186/s12912-024-01945-6 (PMC11055332; doi:10.1186/s12912-024-01945-6)
Supplement: Supplementary file 1 — Supplementary Material 1 [file 12912_2024_1945_MOESM1_ESM.docx]

**Supplementary Material**

# Factors influencing the development of nursing professionalism: A descriptive qualitative study.

**Contents**

S1. Consolidated criteria for reporting qualitative studies (COREQ).

S2. Interview guide.

**S1. Consolidated criteria for reporting qualitative studies (COREQ).**

| **Item** | **Guide questions/description** | **Reported on Page** |
| --- | --- | --- |
| **Domain 1: Research team and reﬂexivity** | | |
| *Personal Characteristics* | | |
| 1. Interviewer/ facilitator | Which author/s conducted the interview or focus group? | Data collection, page 4 |
| 2. Credentials | What were the researcher’s credentials? E.g. PhD, MD | Data collection, page 4 |
| 3. Occupation | What was their occupation at the time of the study? | Data collection, page 4 |
| 4. Gender | Were the researchers male or female? | Female |
| 5. Experience and training | What experience or training did the researcher have? | Data collection, page 4 |
| *Relationship with participants* | | |
| 6. Relationship established | Was a relationship established prior to study commencement? | Participants and settings, page 3-4 |
| 7. Participant knowledge of the interviewer | What did the participants know about the researcher? e.g. personal goals, reasons for doing the research | Participants and settings, page 3-4 |
| 8. Interviewer characteristics | What characteristics were reported about the interviewer/facilitator? e.g. Bias, assumptions, reasons and interests in the research topic | Participants and settings, page 3-4 |
| **Domain 2: study design** | | |
| *Theoretical framework* | | |
| 9. Methodological orientation and Theory | What methodological orientation was stated to underpin the study? e.g. grounded theory, discourse analysis, ethnography, phenomenology, content analysis | Method/Design, page 3 |
| *Participant selection* | | |
| 10. Sampling | How were participants selected? e.g. purposive, convenience, consecutive, snowball | Participants and settings, page 3-4 |
| 11. Method of approach | How were participants approached? e.g. face-to-face, telephone, mail, email | Participants and settings, page 3-4 |
| 12. Sample size | How many participants were in the study? | Findings, page 5 |
| 13. Non-participation | How many people refused to participate or dropped out? Reasons? | N/A |
| *Setting* | | |
| 14. Setting of data collection | Where was the data collected? e.g. home, clinic, workplace | Data collection, page 4 |
| 15. Presence of non-participants | Was anyone else present besides the participants and researchers? | N/A |
| 16. Description of sample | What are the important characteristics of the sample? e.g. demographic data, date | Findings, page 5  &Table 1 |
| *Data collection* | | |
| 17. Interview guide | Were questions, prompts, guides provided by the authors? Was it pilot tested? | Data collection, page 4 |
| 18. Repeat interviews | Were repeat interviews carried out? If yes, how many? | N/A |
| 19. Audio/visual recording | Did the research use audio or visual recording to collect the data? | Data collection, page 4 |
| 20. Field notes | Were ﬁeld notes made during and/or after the interview or focus group? | Data collection, page 4 |
| 21. Duration | What was the duration of the interviews or focus group? | Data collection, page 4 |
| 22. Data saturation | Was data saturation discussed? | N/A |
| 23. Transcripts returned | Were transcripts returned to participants for comment and/or correction? | N/A |
| **Domain 3: analysis and ﬁndings** | | |
| *Data analysis* | | |
| 24. Number of data coders | How many data coders coded the data? | N/A |
| 25. Description of the coding tree | Did authors provide a description of the coding tree? | Fig.1 |
| 26. Derivation of themes | Were themes identiﬁed in advance or derived from the data? | Data analysis, page 4-5 |
| 27. Software | What software, if applicable, was used to manage the data? | N/A |
| 28. Participant checking | Did participants provide feedback on the ﬁndings? | Data collection, page 4 |
| *Reporting* | | |
| 29. Quotations presented | Were participant quotations presented to illustrate the themes/ﬁndings? Was each quotation identiﬁed? e.g. participant number | Findings, pages 5-10 |
| 30. Data and ﬁndings consistent | Was there consistency between the data presented and the ﬁndings? | Findings & Discussion, pages 11-12 |
| 31. Clarity of major themes | Were major themes clearly presented in the ﬁndings? | Findings, pages 5-10 |
| 32. Clarity of minor themes | Is there a description of diverse cases or discussion of minor themes? | Findings, pages 5-10 |

**S2. Interview guide.**

**Introduction**

A short explanation of the study aims, background, and data processing.

Request permission for audio recording.

**Central questions**

| Interview guide for nurse department managers: |
| --- |
| (1) Please describe how you believe nursing professionalism is developed in your work environment. What factors contribute to its formation? |
| (2)What factors do you consider crucial for promoting the development of nursing professionalism? Are there any factors that hinder the growth of nursing professionalism? |
| (3)What factors, in your opinion, can help sustain a nurse's nursing professionalism? |
| (4)From your perspective, what strategies do nursing department managers employ to enhance nursing professionalism? |

| Interview guide for nurse managers: |
| --- |
| (1) Please describe how you believe nursing professionalism is developed in your work environment. What factors contribute to its formation? |
| (2)What factors do you consider crucial for promoting the development of nursing professionalism? Are there any factors that hinder the growth of nursing professionalism? |
| (3)What factors, in your opinion, can help sustain a nurse's nursing professionalism? |
| (4)From your perspective, what strategies do nursing managers employ to enhance nursing professionalism? |

| Interview guide for registered nurses: |
| --- |
| (1) How do you believe nursing professionalism is formed in your nursing career? What factors contribute to it? |
| (2) What do you consider the crucial factors in promoting the development of nursing professionalism? What factors might hinder its growth? |
| (3) What factors, in your opinion, help maintain a nurse's nursing professionalism? |
| (4) What measures would you like managers to take to enhance nursing professionalism among nurses? |

**Summary and ending**

Ask if there are any other thoughts or insights around this topic.

Fill out the socio-demographic form, including age, education, and years of working experience as a nurse.
